# Spontaneous bone regeneration after surgical extraction of a horizontally impacted mandibular third molar: a retrospective panoramic radiograph analysis

**DOI:** 10.1186/s40902-018-0187-8

**Published:** 2019-01-30

**Authors:** Eugene Kim, Mi Young Eo, Truc Thi Hoang Nguyen, Hoon Joo Yang, Hoon Myoung, Soung Min Kim

**Affiliations:** Department of Oral and Maxillofacial Surgery, Dental Research Institute, School of Dentistry, Seoul National University, 101 Daehak-ro, Jongno-gu, Seoul, 110-768 South Korea

**Keywords:** Impacted third molar, Vertical impaction, Radiographic infrabony defect (RID), Pederson’s difficulty index (DI), Panoramic radiograph

## Abstract

**Background:**

The mandibular third molar (M3) is typically the last permanent tooth to erupt because of insufficient space and thick soft tissues covering its surface. Problems such as alveolar bone loss, development of a periodontal pocket, exposure of cementum, gingival recession, and dental caries can be found in the adjacent second molars (M2) following M3 extraction. The specific aims of the study were to assess the amount and rate of bone regeneration on the distal surface of M2 and to evaluate the aspects of bone regeneration in terms of varying degree of impaction.

**Methods:**

Four series of panoramic radiographic images were obtained from the selected cases, including images from the first visit, immediately after extraction, 6 weeks, and 6 months after extraction. ImageJ software® (NIH, USA) was used to measure linear distance from the region of interest to the distal root of the adjacent M2. Radiographic infrabony defect (RID) values were calculated from the measured radiographic bone height and cementoenamel junction with distortion compensation. Repeated measures of analysis of variance and one-way analysis of variance were conducted to analyze the statistical significant difference between RID and time, and a Spearman correlation test was conducted to assess the relationship between Pederson’s difficulty index (DI) and RID.

**Results:**

A large RID (> 6 mm) can be reduced gradually and consistently over time. More than half of the samples recovered nearly to their normal healthy condition (RID ≤ 3 mm) by the 6-month follow-up. DI affected the first 6 weeks of post-extraction period and only showed a significant positive correlation with respect to the difference between baseline and final RID.

**Conclusions:**

Additional treatments on M2 for a minimum of 6 months after an M3 extraction could be recommended. Although DI may affect bone regeneration during the early healing period, further study is required to elucidate any possible factors associated with the healing process. The DI does not cause any long-term adverse effects on bone regeneration after surgical extraction.

## Background

The mandibular third molar (M3) typically erupts last among the permanent teeth due to the lack of available space and thick soft tissues covering its surface [1]. In many cases, impacted M3s require surgical procedures including alveoloplasty and tooth hemisection. Some clinical research has focused on the classification method for these impacted M3s, and Pell and Gregory classification [2] is still considered one of the most effective methods. This classification categorizes M3 based on the relative positions of the ramus of the mandible and the occlusal surface of the adjacent M2 [3, 4]. Statistically, M3 impaction occurs at a high rate of 66%, and a study of 3799 patients over the age of 25 reported that horizontal impaction was most prevalent among angulation types [3, 5]. Among the lesions associated with impacted M3, dental caries occurs in the mandible three times more frequently than in the maxilla [3]. One study found that the incidence of dental caries in the distal surface of M2 associated with M3 was 37.5%, most of which occurred in Pell and Gregory class I and position B [6, 7].

Impacted M3 often causes suppurative inflammation such as chronic periodontitis and odontogenic cysts [1]. In addition, when M3 is extracted, bone absorption, periodontal pocket formation, cementum exposure, and gingival recession may occur in the adjacent second molar [8]. Several studies comparing groups with and without M3 extraction have found significant periodontal tissue destruction at the distal aspect of M2, including increase in probing depth and radiographic alveolar bone loss [9]. Previous retrospective studies with a follow-up of more than 2 years reported that surgical extraction leaves deep infrabony defects but superior bone regeneration capacity in younger age groups [10–12].

Most M3-associated lesions can occur in various forms on the distal surface of M2 and often require additional treatments. For most conservative and periodontal therapies, bone regeneration within the extraction socket should be completed in advance. However, there is a lack of clinical guidance and evidence for the optimal timing of treatment considering bone regeneration of the distal aspect of M2 after extraction of M3. The purpose of this study is to compare and analyze the degree of bone regeneration with respect to time and impaction depth in the extraction socket of mandibular third molars in reference to the distal aspect of adjacent second molars using panoramic radiography.

## Patients and methods

### Sample selection

#### Data acquisition

Among the patients who visited the Department of Oral and Maxillofacial Surgery at Seoul National University (SNUDH) between January 2014 and March 2018, those with impacted mandibular third molars were identified using the Electronic Medical Record (EMR) and Ordering Communication System (OCS). To standardize the operative procedure and minimize procedural discrepancies, all surgeries were performed by a single surgeon (SMK). Based on primary classification criteria, a total of 1674 medical records corresponding to the disease code K01.173 (impacted teeth of mandibular molar, third) were obtained. The study protocol and access to patient records were approved by the Institutional Review Board of SMK (S-D*****).

#### Pell and Gregory classification and Pederson’s difficulty index

In the Pell and Gregory classification, the position of the M3 is indicated as class I, II, or III in relation to the mandibular second molar and as position A, B, or C in relation to the occlusal surface of the adjacent M2. For class I, there is sufficient space to accommodate the mesiodistal width between M2 and the ramus of the mandible. In class II, there is no enough space to accommodate the mesiodistal width of M3, and M3 is positioned completely within the ramus of the mandible. In terms of impaction depth, position A is when the uppermost point of M3 is located at or above the occlusal surface of M2. The uppermost point of M3 is located between the occlusal surface and cervical line of M2 in position B, and the point is located below the cervical line in position C [2, 4]. Pederson’s difficulty index (DI) incorporates the angulation of M3 in addition to the Pell and Gregory classification (Table 1). The DI assigns 1, 2, and 3 points for positions A, B, and C, respectively, and 1, 2, and 3 points for classes I, II, and II. In this study, only horizontally impacted M3s were collected, so two points are added to the DI for calculation. As a result, a DI can be obtained by summing the scores from Pell and Gregory classification and the angulation assessment. Scores of 3 or 4 points can be categorized as minimally difficult, 5 to 7 points as moderately difficult, and 7 to 10 points as very difficult [2, 4].

**Table 1:** Pederson’s difficulty index (DI)

Classification		Value
Spatial relationship	Mesioangualr	1
	Horizontal	2
	Vertical	3
	Distoangular	4
Depth	Position A	1
	Position B	2
	Position C	3
Ramus relationship/space available	Class I	1
	Class II	2
	Class III	3
Difficulty Index	Minimally difficult	3–4
	Moderately difficult	5–7
	Very difficult	8–10

#### Inclusion criteria

Patient profiles were obtained regardless of age or sex. Patients who had undergone surgical extraction of M3 must have undergone recorded panoramic images at the first visit, immediately after extraction (or within 7 days), at a 6-week follow-up, and at a 6-month follow-up. The selection criteria only included horizontally impacted M3 and those corresponding to classes II and III and positions A, B, and C based on the Pell and Gregory classification. Patients with scores between 5 and 8 were qualified for this study. If both impacted M3s of the same patient satisfied the selection criteria, they were independently analyzed and treated as two discrete samples.

#### Exclusion criteria

Patients with jaw-related diseases, systemic diseases directly affecting bone healing, necrosis of the jaw, or a history of bisphosphonate use, head and neck radiation therapy, chemotherapy, or definite periapical lesions were excluded from the study. Patients who had a large subgingival restoration or who did not have M2 were also excluded. In addition, EMR showing postoperative complications that could delay wound healing were excluded, as were patients for whom a panoramic image was not taken at each follow-up visit.

#### Screening sequence

**Fig. 1:**
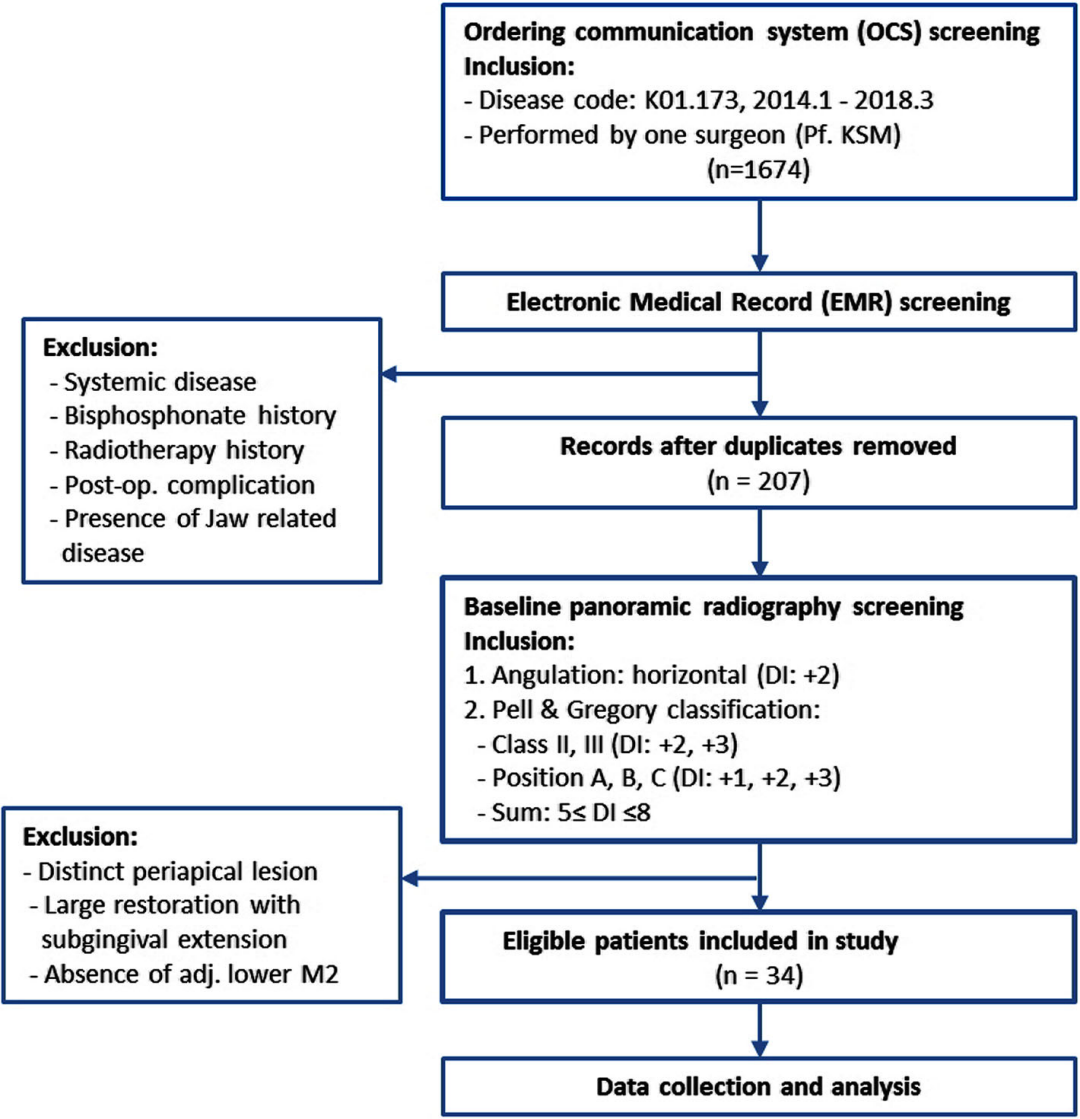
Screening sequence using OCS, EMR, and panoramic radiograph

All screening procedures were performed by a single observer, and the radiographic readings were totally dependent on the observer’s reading skill (Fig. 1).

##### OCS screening

Based on the primary selection criteria, a total of 1674 medical records corresponding to the disease code K01.173 (impacted teeth of mandibular molar, third) were obtained.

##### EMR screening

The exclusion criteria of systemic disease, bisphosphonate history, radiotherapy history, jaw-related disease, and postoperative complication were applied to the 1674 cases using EMRs. Furthermore, because the primary selection was based on outpatient records, patients who had multiple visits with multiple records were combined into a single entry. A total of 207 patients was selected based on the secondary selection criteria.

##### Panoramic radiography screening

Based on panoramic radiographs, patients who did not meet the exclusion criterion were selected based on the following three inclusion criteria. First, only horizontal impaction M3 cases were included regardless of Pell and Gregory class. Then, based on Pell and Gregory classification, teeth were assigned to class II if there was insufficient space between M2 and the ramus and to class III if M3 was located within the ramus. Therefore, class I was excluded, and all M3 depth positions (position A, B, C) were selected. Finally, Pell and Gregory classification and angulation were used to calculate DI values, resulting in a DI ranging from 5 to 8 (Table 1). Preoperative removal of an adjacent M2 was excluded, as was any sign of a definite periapical lesion. A sample was excluded in cases of large restorations on M2 that contained a subgingival margin.

### Study methods

#### Radiographic analysis

Panoramic radiographs were analyzed in reference to the method shown in the study of Faria et al. [13]

##### Panoramic radiograph measurements

**Fig. 2:**
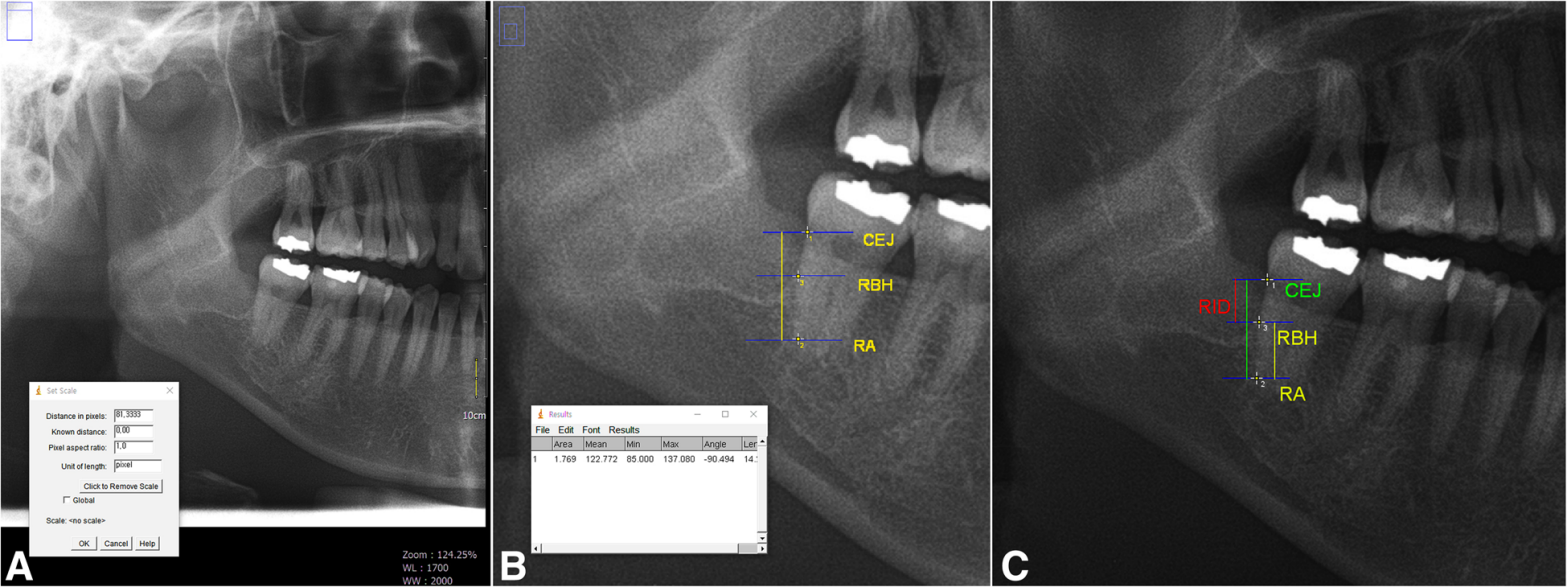
Panoramic radiograph screening using imageJ® software. **a** The process of setting the distance per pixel unit using the scale tool in the ImageJ® software and the ruler in the panoramic image. **b** The process of measuring the desired linear distance in the region of interest (ROI) using the set scale value. **c** Reference points and radiographic variables: (1) CEJ; (2) root apex of the distal root (RA); (3) Uppermost intersecting point between distal root of M2 and mesial wall of extraction socket; RBH, Radiographic bone height (yellow line); distance between CEJ and RA (green line); RID, Radiographic infrabony defect (red line)

Radiographic images of selected patients were extracted using INFINITT PACS® (INFINITT Healthcare, Seoul, Korea). To analyze the region of interest (ROI), the image was adjusted and magnified up to 120% using PACS and exported as a jpg file. Radiographic images at baseline, 6 weeks after extraction, and 6 months after extraction were obtained for each individual. For radiographic analysis, the variables were measured and recorded using ImageJ® (NIH, USA) software. For length measurement, the “straight” tool in ImageJ® was used first to set a 10-cm scale ruler from the original panorama image (Fig. 2a). The linear height of the bone within the ROI was then measured based on the scale set above (Fig. 2b).

The variables to be measured in each panorama image were as follows. First, the upper boundary of M2 root was set as the cementoenamel junction (CEJ) and the lower boundary as the root apex of the distal root (RA). The radiographic bone height (RBH) was determined as the distance between the uppermost point, where M2 distal root and the mesial wall of extraction socket intersected, and the RA. In addition, the radiographic infrabony defect (RID) was determined as the distance from the RBH to the CEJ to evaluate bone regeneration within the socket. Using ImageJ®, the linear distance between CEJ and RBH was measured, and RID was calculated as the difference between those two variables (Fig. 2c). Because all variables were measured manually, the same procedures were repeated three times. The average of these values was used as final RID value to increase accuracy and reduce intra-examiner bias. Ultimately, RID0 (the infrabony defect immediately after extraction), RID6W (infrabony defect after 6 weeks), and RID6M (infrabony defect after 6 months) were calculated and recorded following the same protocol.

##### Panorama radiography distortion correction

Because of its unique nature, panoramic radiography was not able to avoid positioning errors and distortions. Therefore, the difference in distortion rate of the images was revised. The distance from CEJ0 (CEJ at baseline) was used as the reference, and the ratios of CEJ0 to CEJ6W and CEJ0 to CEJ6M were calculated. This ratio, referred to as the distortion factor (DF), was applied to RID6W and RID6M to correct for distortion variations in each image. The final revised RID values were recorded and used for further analysis.

$$ \mathrm{DF}6\mathrm{W}=\frac{\mathrm{CEJ}6\mathrm{W}}{\mathrm{CEJ}0} $$

$$ \mathrm{FinalRID}6\mathrm{W}=\mathrm{RID}6\mathrm{W}\times \mathrm{DF}6\mathrm{W} $$

#### Statistical analysis

Statistical analysis was conducted based on the final values obtained from distortion correction.

##### Repeated measures analysis of variance

Analysis of variance (ANOVA) using repeated measures is applied when comparing means in cases with three or more identical members and is commonly used for a repeated measurement of the same member in relation to time or intervention [14]. ANOVA is one of the parametric tests that follow the normal distribution. However, for a practical reason, if the number of samples in a population exceeds 30, a normal distribution is assumed based on the central limit theorem [14–16]. Therefore, the dependent variables RBH and RID were tested for statistically significant differences in values over time, generating an independent variable.

##### One-way ANOVA test

One-way ANOVA involves dependent variables consisting of nominal scales and independent variables above the isometric scales, and these variables are used to compare three or more group means [14–16]. For a given follow-up period, the dependent variable RID was used to determine whether the mean value was significantly different based on the independent variable DI. As mentioned above, normal distribution was assumed under the central limit theorem [14–16].

##### Bivariate correlation analysis

Correlation analysis examines the independence or the relationship between two variables. A Spearman correlation test was performed for nonparametric validation that did not require variables (DI and RID) for population assumptions. If the results showed statistical significance, the correlation coefficient was used to examine the correlation between the two variables [14–16]. Statistical significance was determined at *p* < 0.05 in all cases. All statistical analyses were performed using SPSS ver. 25.0® for Windows (SPSS Inc., Chicago, IL, USA).

## Results

A total of 1674 outpatient admissions was initially selected, and 28 patients (16 men [57%] and 12 women [43%]) were included, with a total of 34 extraction socket samples after final screening. The age of patients ranged from 23 to 57 years, with an average age of 38.7 ± 11.1 years.

### Classification of impacted mandibular third molar (M3)

According to the relationship of M3 and the ramus of the mandible, 23 sockets were classified as class II (67.6%) and 11 sockets as class III (32.4%). With respect to the occlusal plane of adjacent M2, five sockets were classified as position A (14.7%), 14 as position B (41.2%), and 15 as position C (44.1%) (Table 2 a, b). Out of 34 samples, 26 cases (76.5%) were classified as moderately difficult (DI = 5–7) and the remaining eight (23.5%) as very difficult (DI = 8–10) according to the difficulty index (Table 2c).

**Table 2:** Descriptive data for the number of third molars using Pell and Gregory Classification

	Number	Percent
(a) Classification of third molars in relation to the ramus of the mandible		
Class I	0	0
Class II	23	67.6
Class III	11	32.4
Total	34	100.0
(b) Classification of third molars in relation to the relative depth with respect to adjacent M2 occlusal plane		
Position A	5	14.7
Position B	14	41.2
Position C	15	44.1
Total	34	100.0
(c) Classification of third molars in relation to Pederson’s difficulty index (DI)		
DI		
Minimally difficult	0	0
Moderately difficult	26	76.5
Very difficult	8	23.5
Total	34	100.0

Minimally difficult (DI = 3–4); moderately difficult (DI = 5–7); very difficult (DI = 8–10)

### Analysis of bone regeneration over time using radiographic bone height and radiographic infrabony defect

The mean value of RBH0 at the baseline was 6.71 ± 0.22 mm, and the mean RBH6M value was 13.07 ± 1.05 mm in the images at the 6-month follow-ups. Statistical significance was achieved at *p* < 0.01, and there were statistically significant differences between follow-up groups (RBH0, RBH6W, and RBH6M). In addition, the mean RBH value always increased between follow-up periods. During the first 6 weeks after extraction, RBH showed an average increase of 3.30 ± 2.56 mm (*p* < 0.01). Between the 6-week and 6-month follow-ups, RBH increased 3.06 ± 2.30 mm (*p* < 0.01), and there was an increase of 6.36 ± 2.30 mm (*p* < 0.01) during the entire 6-month follow-up period from baseline (Table 2).

After impacted M3 extraction, the mean RID value decreased over time. The mean RID was 9.58 ± 2.25 mm at baseline, 6.41 ± 2.53 mm at 6 weeks after extraction, and 3.21 ± 1.39 mm at 6 months, and there was a statistically significant difference among the follow-up groups (*p* < 0.01). Average RID differences were evaluated between periods. There was a decrease of − 3.17 ± 2.31 mm (*p* < 0.01) during the first 6 weeks after extraction and of − 3.20 ± 2.12 mm between the 6-week and 6-month follow-ups (*p* < 0.01). An average total decrease of − 6.37 ± 2.28 mm in RID during the 6 months after extraction was observed (Table 3).

**Table 3:** Average changes in RBH and RID over time (*n* = 34)

Radiographic variables	Assessments (weeks)	Mean (mm)	SD (mm)	Differences between assessments (mm)	SD (mm)	*P* value*
RBH	0	6.71	0.22	3.30	2.56	.000
	6	10.01	0.26	3.06	2.30	.000
	24	13.07	0.15	6.36	2.41	.000
RID	0	9.58	2.25	− 3.17	2.31	.000
	6	6.41	2.53	− 3.20	2.12	.000
	24	3.21	1.39	− 6.37	2.28	.000

Abbreviations: *RBH* radiographic bone height, *RID* radiographic infrabony defect

*Statistically significant differences over time at *p* < 0.01

The RID was categorized according to ≤ 3 mm, > 3 to ≤ 6 mm, and > 6 mm and showed statistical significance with respect to follow-up period. At baseline, RIDs ≤ 3 mm (0%) were absent, and RIDs > 6 mm (91.2%) were predominant; after 6 months, RIDs > 6 mm decreased to 2.9%, and RIDs ≤ 3 mm increased to 61.8%. In addition, at 6 weeks of follow-up, RIDs > 3 mm to ≤ 6 mm increased from 8.8 to 44.1%, RIDs > 6 mm decreased to 52.9% during the first 6 weeks, and RIDs ≤ 3 mm were 2.9% at 6 months (Table 4).

**Table 4:** Descriptive data for RIDs at each assessment (*n* = 34)

	Assessments (weeks)					
	0		6		24	
RID (mm)	*n*	%	*n*	%	*n*	%
≤ 3	0	0	1	2.9	21	61.8
> 3 to ≤ 6	3	8.8	15	44.1	12	35.3
> 6	31	91.2	18	52.9	1	2.9
Total	34	100	34	100	34	100

### Analysis of bone regeneration using Pederson’s difficulty index

The differences between the RBHs according to assessment period were grouped into three categories. The difference between baseline RBH0 and RBH6W was defined as RBH6W_RBH0, the difference between RBH6W and RBH6M as RBH6M_RBH6W, and the difference between RBH6M and baseline as RBH6M_RBH0. Repeated measures of analysis of variance were used to test the statistical significance (*p* < 0.05). Descriptive data on RBH change showed some bone loss in RBH6W_RBH0 (8.8%) and RBH6M_RBH6W (2.9%). However, when comparing the final evaluation of RBH6M with baseline RBH0, bone gain occurred in all cases (Table 5).

**Table 5:** Descriptive data for differences recorded between RBH values as bone gains and losses between assessments (*n* = 34)

	Assessment period		
	RBH6W_RBH0	RBH6M_RBH6W	RBH6M_RBH0
Bone gains (mm)			
Maximum	7.86	11.24	11.07
Minimum	0.35	0.22	2.09
Mean	3.67	3.18	6.36
Median	3.84	2.94	6.62
Variance	5.63	4.91	5.83
SD	2.37	2.22	2.41
*n*	31	33	34
Bone losses (mm)			
Maximum	− 1.02	− 1.10	–
Minimum	− 0.19	− 1.10	–
Mean	− 0.48	− 1.10	–
Median	− 0.23	− 1.10	–
Variance	0.22	–	–
SD	0.47	–	–
*n*	3	1	0

Abbreviations: *RBH6W_RBH0* difference between the radiographic bone height recorded at 6 months and at baseline, *RBH6M_RBH6W* difference between the radiographic bone height recorded at 6 months and at 6 weeks, *RBH6M_RBH0* difference between the radiographic bone height recorded at 6 months and at baseline

A negative value indicates loss, but the amount of loss is in its absolute value

Initial RID values with respect to DI were 10.91 ± 1.47 mm for a DI score of 8 points, 9.86 ± 2.70 mm for 7 points, 9.17 ± 2.15 mm for 6 points, and 7.78 ± 1.33 mm for 5 points (Table 6). The difference between baseline RID and RID6W was defined as RID6W_RID0, the difference between RID6W and RID6M as RID6M_RID6W, and the difference between RID6M and RID0 as RID6M_RID0. Among the 34 samples, five cases were classified as having DI = 5 points (14.7%), 11 cases as DI = 6 points (32.4%), eight cases as DI = 7 points (29.4%), and eight cases as DI = 8 points (23.5%). For average RID6W_RID0, the greatest RID decrease (− 5.37 ± 2.80 mm) was recorded at a DI score of 8 points. In RID6M_RID6W, the greatest RID decrease (− 4.08 ± 2.45 mm) was recorded at 7 points, while the greatest RID decrease (− 8.22 ± 1.63 mm) was recorded at 8 points in RID6M_RID0. The mean RID differences between the assessments were compared using the DI. RID6W_RID0 showed statistical significance (*p* > 0.05), while RID6M_RID6W and RID6M_RID0 showed no statistical significance (*p* > 0.05) (Table 7).

**Table 6:** Descriptive data for mean RID0 (baseline) according to the difficulty index (DI)

DI	*N*	Minimum	Maximum	Mean	SD
5	5	6.38	9.50	7.78	1.33
6	11	5.24	12.49	9.17	2.15
7	8	5.58	13.51	9.86	2.70
8	5	8.12	12.70	10.91	1.47

**Table 7:** Comparison of mean RID change according to Pederson’s difficulty index (DI)

DI	Assessment		
	RID6W_RID0	RID6M_RID6W	RID6M_RID0
5 (*n* = 5)	− 3.20 ± 1.64^a^	− 2.63 ± 1.44^b^	− 5.83 ± 1.68^b^
6 (*n* = 11)	− 2.56 ± 2.11^a^	− 2.91 ± 2.24^b^	− 5.47 ± 2.43^b^
7 (*n* = 10)	− 2.07 ± 1.09^a^	− 4.08 ± 2.45^b^	− 6.15 ± 2.21^b^
8 (*n* = 8)	− 5.37 ± 2.80^a^	− 2.86 ± 1.86^b^	− 8.22 ± 1.63^b^
*p* value	0.010	0.488	0.053

Abbreviations: *RID6W_RID0* difference between the radiographic infrabony defect recorded at 6 months and at baseline, *RID6M_RID6W* difference between the radiographic infrabony defect recorded at 6 months and at 6 weeks, *RID6M_RID0* difference between the radiographic infrabony defect recorded at 6 months and at baseline

^a)^Statistically significant decrease in mean RID6W compared with baseline (RID0) according to Pederson’s difficulty index (DI) (*p* < 0.05) by one-way ANOVA

^b)^No statistically significant decrease in mean RID change according to DI (*p* > 0.05) by one-way ANOVA

Correlation analysis was performed for DI and RID differences among follow-up periods. The correlation coefficient was 0.222 (*p* > 0.05) in RID6W_RID0, 0.108 (*p* > 0.05) in RID6M_RID6W, and 0.396 (*p* < 0.05) in RID6M_RID0. Only the results of the correlation analysis between RID6M and baseline RID0 during the final evaluation were statistically significant (*p* < 0.05), and a positive correlation was observed (Table 8).

**Table 8:** Correlation analysis of RID change with respect to Pederson’s difficulty index (DI)

Assessments	Spearman’s rho	
	Correlations coefficient	Sig. (two-tailed)
RID6W_RID0	0.222	0.206
RID6M_RID6W	0.108	0.541
RID6M_RID0	0.395^#^	0.021

Abbreviations: *RID6W_RID0*, difference between the radiographic infrabony defect recorded at 6 months and at baseline, *RID6M_RID6W* difference between the radiographic infrabony defect recorded at 6 months and at 6 weeks, *RID6M_RID0* difference between the radiographic infrabony defect recorded at 6 months and at baseline

^#^Correlation is significant at the 0.05 Position (2-tailed)

^#^Note that the difference between RID value is in its absolute value for the statistical analysis

## Discussion

In the Pell and Gregory classification, the position of the third molar is determined by the relationship between the ramus of the mandible and the mandibular adjacent M2. Classes I, II, and III specifies the mesiodistal width between M2 and the ramus, while positions A, B, and C refers to the vertical depth with respect to the M2 occlusal plane. DI scores combine the Pell and Gregory classification and the Winter classification, which defines the angulation of M3. In this study, only horizontally impacted samples were collected; therefore, two points were added equally to each DI score. The difficulty index assigns 1, 2, or 3 points for position A, B, or C, respectively, and 1, 2, or 3 points for class I, II, or II. The final DI score can be obtained by adding the scores of Pell and Gregory classification and Winter classification. A DI score of 3 or 4 points is categorized as minimally difficult, 5 to 7 points as moderately difficult, and 7 to 10 points as very difficult [2, 4].

The samples collected for this study were homogeneous in nature because all were horizontally impacted M3s with a DI score between 5 and 8 points. In addition, the panoramic radiograph images were collected on the basis of patient histories that did not include complications or diseases that may affect bone regeneration. Kugelberg et al. [10–12] reported that bone regeneration after M3 extraction is affected by age and is more likely to occur in younger patients under 25 years of age. However, this study was performed independent of the age of patients (38.7 ± 11.1 years).

In addition to clinical exams, radiographic exams are one of the major determinants of clinical bone regeneration and recovery following M3 extraction [10, 17–19]. Time is an important variable in the analysis of radiographic images and has a direct effect on other measured variables in the image. Many previous retrospective studies focused mainly on bony changes over time after M3 extraction [10, 17–20]. In spite of periapical radiograph is recommended to measure the bone level and bone margin evaluation with panoramic radiograph is not a standard method, the strength of this study was its homogenous collection of horizontally impacted M3s and inclusion of DI as an analyzed variable while focusing on bone regeneration over time.

Panoramic radiography is widely used in routine dental procedures such as implant placement, and it has the advantage of showing surrounding anatomical structures as well as the teeth. However, the panoramic image is magnified and distorted beyond actual size when the patient is out of the focal trough. Even if screened using a variate procedure, panoramic radiograph has an average magnification of 15 to 25% depending on the patient’s position [21]. The magnification rate can be affected by the shape and size of the patient’s jaw and is greatest at the canine and premolar regions and lowest at the third molar region [22, 23]. Therefore, it is difficult to position the patient accurately in the focal trough, even with the help of an aiming light. According to an ideal experimental study, the vertical magnification ratio showed less variation and more consistent results than horizontal magnification ratio [24]. In a study comparing the reliability of cone-beam computed tomography and panoramic radiography, although a vertical overestimation of 0.87 mm occurred as the alveolar process moved 1 mm toward the lingual side, it concluded that such errors are acceptable for clinical use [25].

A reliable and standardized diagnostic method, such as assessment of infrabony defects recovery after M3 extraction, is required to assess bony changes over time. However, many existing studies used various types of images with different measuring tools, and it was difficult to compare the data or results [9, 10, 17, 18, 20, 26]. In this study, the existing method proposed by Faria et al. [13] was employed to minimize deviating from the recent research standards. Bone regeneration after M3 extraction occurred constantly over time. The RID was 9.58 ± 2.25 mm at baseline, 6.41 ± 2.53 mm after 6 weeks, and 3.21 ± 1.39 mm after 6 months. In Faria et al. [13], the initial RID0 was 4.54 ± 1.87 mm, and RID6M was 2.59 ± 1.85 mm. Bone regeneration was 1.40 ± 2.00 mm and 0.56 ± 1.19 mm at 3- and 6-month follow-ups, respectively. Another study by Faria et al. [26] showed a 1.62 ± 2.44 mm recovery of periodontal pocket depth during the first 3 months after extraction, and there was no significant change in pocket depth between the 3-month and 12-month follow-ups. Although there was a difference in RID values, such a difference was considered reasonable in the present study because the samples were all M3s with deep horizontal impaction. In this study, a 3-month follow-up image was not included, but active bone regeneration was observed initially over the short period following extraction.

In case of large extraction sockets, the proportion of RIDs > 6 mm decreased dramatically from 91.2% to 2.9% during the 6-month follow-up period. In addition, 61.8% of infrabony defects recovered nearly to the physiologic condition of RID ≤ 3 mm. An analysis of RBH between evaluation periods showed that a few cases exhibited bone loss in the early stages, but all eventually showed bone gain after the final follow-up of 6 months. Therefore, as with the in vivo study of mongrel dogs, it appears that transient bone loss was caused by osteoclast activity during the early stages of bone remodeling [27, 28]. Because there was considerable individual variation in terms of bone-healing rate, it was difficult to predict the healing progress of a patient at a given time.

More recent research has focused on peripheral bone changes that occur with post-extraction healing. In vivo studies using mongrel dogs showed bone resorption through osteoclast activity during the first 8 weeks after extraction, causing a decrease in vertical height [27]. Although 88% of the extraction socket was replaced with mineralized bone 30 days after extraction, the mineralized tissue decreased to 15% after 180 days, and the bone marrow increased to 85% over time [27, 28]. In actual clinical settings, patients with M3 extraction showed periodontal problems to some extent during the first 3 months of follow-up, but the problems lessened remarkably after 1 year [29]. Another study reported that bone healing did not occur during the first 3 months after extraction, but infrabony defects recovered to their original state after 12 months [13].

The difficulty of impacted mandibular M3 extraction can be influenced by the shape of the tooth, the location within the arch, the depth of impaction, and the angulation of tooth. Above all, impaction depth and angulation are directly related to difficulty in extraction [30]. In this regard, the DI using the Pell and Gregory classification and the Winter angulation classification can play an important role in diagnosis and preoperative planning. Among the total of 34 study samples, five had a DI score of 5, 11 samples were assigned a DI score of 6, 10 samples a DI score of 7, and eight samples a DI score of 8; all were classified as moderately difficult or very difficult. RID differences were analyzed with respect to DI scores, and only the difference between baseline RID and RID6W showed statistical significance (*p* < 0.05). Within the RID6W_RID0, the group with a DI score of 8 had the highest average RID differences (− 5.37 ± 2.80 mm). A difference in the RID value is a measure of the degree of bone regeneration. These differences between RID values represented the amount of bone regeneration, and initial bone regeneration was observed during the early stage of the healing process. However, further study was needed to verify the current results and to reveal the contributing factors that might have affected bone regeneration.

The correlation coefficient between the DI score and the RID difference was only statistically significant in RID6M_RID0 (*p* < 0.05), which showed a relatively low positive correlation coefficient of 0.396. Thus, patients with higher initial DI scores would have a higher absolute amount of bone regeneration. In this context, the samples used in the present study also showed greater bone regeneration with a deeper initial RID0 and higher DI score, indicating a positive correlation. As a result, extraction difficulty had no significant effect on initial bone regeneration, although it might affect final bone regeneration, and the increase in initial RID could result in greater bone regeneration.

It is important to obtain standardized measurements and images in radiographic analysis as in the present study. Several studies mentioned that it is difficult to standardize panoramic images [21, 24]. In Faria et al. [13], a modified intraoral radiography device was used to reduce and standardize the error between images. However, in the present study, it was impossible to avoid distortions from characteristics of panoramic images. Instead, to compensate for the difference in distortion rate between images, a DF was calculated and applied to the RID values. Furthermore, to reduce human error and intra-examiner bias in analyzing panoramic images, it is necessary to use a radiopaque indicator, such as the dental probe used by Faria et al. [13]. Without these devices, the present study was left with some limitations: pre-extraction RID could not be measured due to superimposition of teeth. Lastly, unlike most studies focusing on bone regeneration, which included a minimum of 1-year follow-up, the present study only had a 6-month follow-up period because of limitation in the research settings.

Large RIDs (> 6 mm) that developed immediately after extraction constantly decreased over time and recovered to a normal range (RID ≤ 3 mm) in more than half of the cases after 6 months of extraction. Although bone regeneration after tooth extraction occurred actively throughout the first 6 months, extraction difficulty was significantly affected within the first 6 weeks. Correlation analysis between extraction difficulty and bone regeneration showed that the increase in infrabony defects may lead to enhanced bone healing in the long term. While DI did not affect long-term bone healing from 6 weeks to 6 months, it did affect initial bone regeneration; therefore, further study will be needed to determine the specific factors associated with the initial bone-healing process.

As a result, if additional treatments of an adjacent M2 are required after M3 extraction, it is recommended that clinicians do not proceed with further treatment during the first 6 months after extraction. However, because bone regeneration patterns, rate, and recovery ability vary greatly among individuals, it is difficult to predict the absolute stage of bone regeneration in a patient. Clinicians must perform clinical and radiographic exams before proceeding with further treatments. Extraction difficulty appears to affect bone regeneration, but further research is needed on the related factors.

## Conclusions

It could be recommended that clinicians do not proceed with further treatment of adjacent M2 during the first 6 months after M3 extraction. Clinicians must perform clinical and radiographic exams before proceeding with further treatments, and especially panoramic analysis could be helpful for the related factor considerations on bone regenerations.

## Abbreviations

CEJ: Cementoenamel junction
DF: Distortion factor
DI: Pederson’s difficulty index
EMR: Electronic Medical Record
OCS: Ordering Communication System
RA: Distal root
RBH: Radiographic bone height
RID: Radiographic infrabony defect
ROI: Region of interest
